# Advancing cross-domain integration and semantic technologies: proceedings of the 19th international symposium on integrative bioinformatics

**DOI:** 10.1515/jib-2026-0019

**Published:** 2026-08-11

**Authors:** Mary-Ann Ebeling, Matthias Lange, Stephan Weise, Uwe Scholz

**Affiliations:** Department Breeding Research, Leibniz Institute of Plant Genetics and Crop Plant Research (IPK), Seeland, Germany; Department Genebank, Leibniz Institute of Plant Genetics and Crop Plant Research (IPK), Seeland, Germany

**Keywords:** integrative bioinformatics, FAIR principles, research data management (RDM), cross-omics integration, open science

## Abstract

This special issue of the *Journal of Integrative Bioinformatics* presents proceedings from the 19th International Symposium on Integrative Bioinformatics, which was held 10–12 September 2025. The featured contributions address critical bottlenecks in life science data management, specifically focusing on the practical implementation of FAIR (Findable, Accessible, Interoperable, and Reusable) principles as an essential prerequisite for data integration and data interoperability. Comprising nine peer-reviewed articles, the issue highlights methodological advancements in cross-omics integration, semantic technologies for machine interpretability, and the deployment of robust digital infrastructures like RO-Crate. By bridging technical innovation with community-driven data stewardship, this collection provides actionable frameworks to modernize the research data lifecycle and ensure computational reproducibility across the life sciences.

## 1 Introduction

High-throughput technologies across the life sciences and, for example, in plant research continue to generate immense volumes of empirical data. However, the inherent diversity of these heterogeneous data domains and the fragmented nature of current repositories create substantial bottlenecks for researchers. Managing and publishing these datasets in strict accordance with FAIR (Findable, Accessible, Interoperable, and Reusable) principles [1] remains a tough challenge. Furthermore, the capacity to integrate data across multiple domains serves as a necessary prerequisite for deploying advanced analytical frameworks, including generative artificial intelligence and comprehensive knowledge graphs. To address these systemic hurdles and coordinate solutions aligned with national and international initiatives – such as NFDI, ELIXIR, BioFAIR, and ARDC – the 19th International Symposium on Integrative Bioinformatics (GRC-IB2025) convened at the Leibniz Institute of Plant Genetics and Crop Plant Research. This event gathered an international cohort of scientists to evaluate current bottlenecks and advance the state of integrative data management. For more details see: https://www.imbio.de/ib2025.

## 2 Core themes

The conference program addressed the complete data lifecycle, highlighting distinct methodological shifts and infrastructural requirements. Participants examined data governance and open science, focusing on the practical implementation of FAIR principles. Discussions targeted both technical mechanisms – such as secure data containers and the merging of experimental and computational provenance via RO-Crate [2] – and organizational shifts toward human-centric, process-oriented data stewardship models. Methodological advancements featured heavily in cross-omics modeling and data science analytics. Researchers presented refined bioinformatic workflows for biosynthetic pathway prediction, standardized metabolic models, and accessible platforms designed to democratize data analysis.

A critical consensus emerged regarding the necessity of semantic technologies to ensure machine interpretability; researchers demonstrated how AI-driven knowledge graphs and automated text-mining pipelines structure complex datasets down to the level of individual data points. Finally, the sessions underscored that robust digital infrastructures – ranging from field-data mobile applications to “Digital Twin” frameworks – must integrate with active community engagement. Structured training networks and the deployment of FAIR Digital Objects [3] are essential to instigate cultural change and ensure computational reproducibility across scientific communities.

## 3 Introduction to the special issue

Drawing upon the discussions at GRC-IB2025, the organizers selected nine contributions for this special issue of the *Journal of Integrative Bioinformatics*. These peer-reviewed articles provide specific solutions to pressing data integration and analytics challenges by bridging technical innovation with community-driven adoption. Ranging from molecular sequence analysis and metabolomics pipelines to scalable data containers, workflow provenance standards, and cultural change frameworks, the curated papers collectively advance FAIR principles as both a technical and social endeavor.

### 3.1 Computational biology and analytical workflows

Aligned with the conference’s focus on integrative modeling and cross-omics platforms, these papers tackle the foundational challenge of extracting biological meaning from complex datasets through standardized, reproducible workflows. Frigerio details the GetFeatistics R-package, which integrates calibration-curve quantification, quality control-based feature filtering, and mixed-effects and Tobit regression models into a single documented pipeline to facilitate both exploratory studies and large-scale epidemiological applications [4]. To support complex multi-omics analyses, Dörpholz et al. showcase how the Annotated Research Context (ARC) framework enables modular workflow execution and standardized metadata annotation for generating a single-cell cross-omics transcriptome atlas [5].

### 3.2 FAIR data infrastructure and standards

Directly engaging with the conference sessions on open science, FAIR digital objects, and data stewardship, four papers propose novel technical mechanisms. Schlegel et al. introduce Pithos, a next-generation container format combining content-defined chunking, smart compression, convergent encryption, and embedded semantic metadata [6]. Ott et al. discuss the ARC Workflow Run RO-Crate profile, which unifies computational and laboratory provenance within a single queryable metadata graph to enable a homogenized description of the entire research lifecycle – from biological source material to the final visualization of result statistics [7]. Additionally, Weil et al. introduce Data Fragment Selectors (DFS) and the Datamap approach, enabling format-agnostic annotation at sub-file granularity to establish precise, machine-actionable links between process metadata and primary data [8]. Further advancing data annotation, Quadros et al. describe a text-mining pipeline with expert supervision designed to support and scale annotation processes for scientific information systems [9]. Finally, Söchting et al. present Pheno-App 2.0 to address the FAIR capture of plant phenotypic data. This open-source Android application integrates directly with institutional database systems to link data governance with practical, field-level acquisition, providing an important tool to support genebank experts in the documentation and conservation of plant genetic resources [10].

### 3.3 Community engagement, service monitoring, and cultural change

Reflecting the conference’s emphasis on democratized research frameworks, these papers demonstrate that technical infrastructure requires active community adoption. Sennhenn et al. present FAIRagro’s Community Engagement and Empowerment Cycle, a four-phase framework (Identify, Translate, Communicate, Anchor) grounded in participatory principles, confirming that structured community engagement is a prerequisite for systemic change [11]. Finally, Feser et al. present the Scorpion tool, a key performance indicator (KPI) monitoring platform that harmonizes performance metrics across federated service portfolios; this tool enables service providers to retain control over their metrics while supporting centralized reporting and strengthening accountability toward funding agencies such as DFG/NFDI within infrastructures like NFDI4Biodiversity and de.NBI/ELIXIR-DE [12].

Taken together, these nine contributions chart a coherent progression from data analysis through interoperable infrastructure to community-driven adoption, directly mirroring the GRC-IB2025 vision to systematically advance integrative bioinformatics.

## 4 Keynote contributions

In addition to the thematic sessions, the symposium featured seven keynote lectures addressing the intersection of data governance, semantic technologies, and digital infrastructures.

**Sarah Dyer (EMBL-EBI, UK)** presented Ensembl as a comprehensive platform for integrating public genomic data. She demonstrated how the resource harmonizes genetic variation and comparative genomics data across eukaryotic species to support structural variation analysis and evolutionary research.

**Carolyn Lawrence-Dill (Colorado State University, USA)** provided a retrospective on plant bioinformatics, emphasizing the practical application of computational tools to predict gene function and phenotype. Her talk underscored the necessity of interdisciplinary collaboration and structured training to support data-driven agricultural sciences.

**Josh Moore (German BioImaging – Society for Microscopy and Image Analysis e.V., Germany)** traced the evolution of bioimaging infrastructure to manage increasingly dense, multidimensional data. He detailed the shift from restrictive proprietary file formats to cloud-native, scalable solutions like OME-Zarr, emphasizing the need for structured, machine-readable metadata.

**Timo Mühlhaus (RPTU Kaiserslautern-Landau, Germany)** proposed a declarative, process-centric approach to data modeling. He introduced the Annotated Research Context (ARC) framework as a method to encode data provenance, linking experimental metadata with computational workflows to enable semantic inference across diverse scientific contexts.

**Taner Z. Sen (USDA-ARS and University of California, Berkeley, USA)** outlined the management of the GrainGenes database. He illustrated the necessity of metadata standards in managing agricultural resources and discussed the application of machine learning models to extract actionable biological knowledge from heterogeneous genomic datasets.

**Nils Stein (Leibniz Institute of Plant Genetics and Crop Plant Research and Martin-Luther-University Halle-Wittenberg, Germany)** detailed the application of pan-genome approaches to explore structural genome diversity in major cereals. He demonstrated how genomic insights integrate directly with genebank resources to advance the understanding of crop plant genetics and support biodiversity conservation.

**Ulrike Wittig (Heidelberg Institute for Theoretical Studies, Germany)** examined the transition from manual literature curation to structured FAIR data. She highlighted resources such as SABIO-RK and FAIRDOM-SEEK, which provide frameworks for standardizing biochemical networks and managing project-wide metabolic models.

## 5 Conclusion & outlook

The momentum generated at GRC-IB2025 indicates a clear trajectory for the life sciences: the future of integrative bioinformatics relies on merging rigorous semantic standardization with scalable digital infrastructures. As biological models grow more complex and artificial intelligence applications become standard practice, the scientific community must consistently prioritize machine-actionable FAIR principles and collaborative data stewardship.

Continuing this trajectory, the next Integrative Bioinformatics Conference will convene again at the Leibniz Institute of Plant Genetics and Crop Plant Research from September 2–4, 2026. Hosted within the frame of the NFDI for Life Sciences Conference, this upcoming symposium explicitly targets the architects and developers of the life science data ecosystem. It will gather bioinformaticians, data scientists, research software engineers, training experts, and research data management (RDM) specialists across the entire data lifecycle with the primary objective to drive professional exchange, standardization, and technological development. Plenary sessions and discussions will focus beyond application, targeting the development of novel algorithms and tools, the integration of artificial intelligence into RDM practices, and the rigorous implementation of FAIR data standards. We encourage the community to utilize this exchange to present new infrastructure services, debate data stewardship methodologies, and actively shape the technological landscape of the life sciences. For more details visit https://meetings.ipk-gatersleben.de/nfdi4LS-IB2026/.
